# Moderate Methionine Reduction Alleviates Lipopolysaccharide-Induced Stress in Broiler Chickens by Enhancing Antioxidant Pathways

**DOI:** 10.3390/ani16071069

**Published:** 2026-04-01

**Authors:** Jin Niu, Yuanyang Dong, Meimei Du, Zhihao Zhang, Jianing Fu, Yipeng Zhao, Qiyue Kang, Miaomiao Han, Chenxuan Huang, Xiangdong Guo, Zhenguo Yan, Zhiqiang Miao, Jianhui Li

**Affiliations:** 1 College of Animal Sciences, Shanxi Agricultural University, Taigu, Jinzhong 030801, China; niujin302@163.com (J.N.); yuanyangdong@sxau.edu.cn (Y.D.); 17631513615@163.com (M.D.); zzhaia@163.com (Z.Z.); 18463991106@163.com (J.F.); m15294991070@163.com (Y.Z.); 17803508408@163.com (Q.K.); hanmiao2019@sxau.edu.cn (M.H.); huangchenxuanvip@163.com (C.H.); 2 Fenxi Xinxing Hope Liuhe Food Co., Ltd., Linfen 031500, China; 15135718577@163.com (X.G.); yanzg@zhongxinfood.com (Z.Y.)

**Keywords:** antioxidant capacity, broiler, lipopolysaccharide, methionine metabolism, methionine supplementation, methionine reduction

## Abstract

Methionine (Met), an essential amino acid involved in antioxidant defense and immune regulation in all vertebrates, may play a critical role in modulating acute immune stress responses; however, whether methionine reduction or supplementation in broilers is more beneficial during acute immune challenge remains unclear. To address this gap, this study compared the effects of dietary methionine reduction and supplementation on growth performance, antioxidant status, immune responses, and methionine metabolism in broilers subjected to lipopolysaccharide (LPS) challenge. In total, 504 one-day-old male broilers were assigned to four treatment groups: control (CON, 0.55%, marked as 100%Met), lipopolysaccharide-challenged (LPS, 0.55%, marked as 100%Met), methionine-restricted with LPS challenge (MR + LPS, 0.35%, marked as 60%Met), and methionine-supplemented with LPS challenge (MS + LPS, 0.75%, marked as 140%Met) groups. The experiment lasted for 21 days. On days 17, 19, and 21, broilers in the LPS-stimulated groups received intraperitoneal injections of LPS at 1 mg/kg body weight. Methionine restriction increased the feed conversion ratio before challenge, whereas average daily gain decreased in both LPS and MS + LPS groups during the challenge. Serum alanine aminotransferase, aspartate transaminase, LPS, corticosterone, interleukin-1β, interleukin-6, tumor necrosis factor-α, and hepatic malondialdehyde levels were reduced in the MR + LPS group compared with the LPS group (*p* < 0.05), whereas interleukin-10, antioxidant enzyme activities, total antioxidant capacity, and hepatic expression of antioxidant- and sulfur-metabolism-related genes were increased (*p* < 0.05). These findings indicate that moderate methionine restriction during acute immune stress enhances antioxidant capacity, alleviates hepatic burden, and supports metabolic stability in broilers.

## Simple Summary

Modern poultry production typically involves raising large numbers of birds in confined spaces, which can increase the risk of bacterial infection and cause excessive inflammation and oxidative stress. Oxidative stress occurs when harmful molecules damage cells faster than the body can neutralize them, weakening the immune system and reducing growth. Methionine is an essential amino acid that chickens must obtain from their diet; it supports growth, liver health, and the body’s natural antioxidant and immune defenses. However, it is unclear whether reducing or increasing methionine intake is more beneficial when birds face sudden immune stress. In this study, we compared the effects of normal, reduced, and increased dietary methionine levels in broiler chickens exposed to a bacterial stress challenge throughout the experimental period. We found that temporarily reducing methionine to 60% of the standard level during the acute stress period improved antioxidant defenses, reduced signs of liver strain and inflammation, and helped maintain metabolic balance. These findings suggest that adjusting methionine levels during short-term immune stress can improve poultry health and production efficiency, offering practical guidance for more sustainable and resilient poultry farming.

## 1. Introduction

Intensive high-density poultry rearing can cause increased susceptibility to bacterial infections in poultry through excessive inflammatory and oxidative stress, increasing susceptibility to bacterial infections and impairing production efficiency and carcass quality [1,2]. Lipopolysaccharide (LPS), a major component of Gram-negative bacteria, is widely used in research as a trigger for immune and oxidative stress [3]. LPS triggers oxidative stress by overwhelming antioxidant defenses, leading to excess reactive oxygen species (ROS) generation and oxidative damage to biomolecules [4]. In poultry, LPS exposure not only reduces systemic antioxidant capacity but also activates inflammatory pathways [5]. Studies employing acute post-LPS sampling designs consistently demonstrate pronounced oxidative and immune disturbances in broilers [6]. These findings highlight the need for nutritional strategies to mitigate acute immune stress.

Methionine (Met) is a nutritionally essential and limiting amino acid in poultry diets, which plays a critical role in protein synthesis and metabolic regulation [7,8]. It is among the first amino acids included in feed formulation [9]. Beyond its role in protein synthesis, methionine exhibits antioxidant and immunostimulatory effects [10], and its metabolism produces key metabolites in the methylation cycle, including S-adenosylmethionine (SAM), S-adenosylhomocysteine (SAH), homocysteine, and cysteine. Notably, these metabolites are essential for regulating intracellular methylation reactions, modulating antioxidant capacity, and influencing immune function [11]. Dietary methionine supply is largely associated with the regulation of immune function [12]. Modulating dietary methionine levels, either through reduction or supplementation, has been shown to confer benefits under a range of physiological and stress conditions. For example, reducing methionine levels can alleviate ischemia–reperfusion-induced myocardial injury in mice by activating the cystathionine-γ-lyase stress pathway [13]. Dietary methionine reduction in mice also significantly reduces circulating ROS and inflammatory mediators while improving physical performance [14]. Under LPS challenge, methionine reduction enhances antioxidant defense and suppresses excessive inflammatory signaling in broilers, thereby alleviating LPS-induced liver injury [15]. However, during periods of rapid growth and immune system development, adequate methionine supply is equally essential for maintaining normal physiological function. Appropriate methionine supplementation during early developmental stages has been shown to markedly improve growth performance and antioxidant capacity in broilers [16]. During *Eimeria* infection, broilers fed L-Met exhibited improved growth performance, intestinal integrity, and antioxidant capacity [17]. Furthermore, methionine supplementation enhances immune cell proliferative responses to trinitrophenyl-LPS stimulation and increases the number of immunoglobulin M-secreting cells, indicating improved humoral immune responses [18]. Related evidence also suggests that methionine-supplemented diets can ameliorate age-related deterioration of intestinal function in mice [19].

Despite this, systematic comparisons of methionine reduction and supplementation under immune stress conditions remain limited in broilers. In particular, the optimal methionine strategy under LPS challenge and its associated metabolic regulatory mechanisms are not well understood. To address this, the present study compared the effects of methionine reduction and supplementation on growth performance, antioxidant capacity, immune responses, and methionine metabolism in broilers subjected to LPS-induced immune stress, with the aim of providing a scientific basis for optimizing dietary methionine strategies in poultry production.

## 2. Materials and Methods

### 2.1. Animal Care

All experimental procedures and animal care were approved by the Institutional Animal Care and Use Committee (IACUC) of Shanxi Agricultural University (SXAU), Taigu, China, under protocol SXAU-EAW-2022Po.SD.01129001 (approved on 31 December 2022).

### 2.2. Animals and Experimental Design

A total of 504 one-day-old male Arbor Acres broilers with an initial body weight of 38.89 ± 0.50 g were randomly assigned to four treatment groups (six replicates per group, with 21 chicks per replicate). The replicate cage was considered the experimental unit for growth performance analysis. The cage area was 0.7 m^2^, with 21 chickens per cage. Broiler breeders were provided by Fenxi Xinxing Hope Liuhe Food Co., Ltd. (Linfen, China). The groups were as follows: (1) control group (CON): birds received a basal diet (0.55%; marked as 100%Met) without LPS challenge; (2) LPS-challenged group (LPS): birds received a basal diet (0.55%; marked as 100%Met) but were exposed to LPS; (3) methionine-restricted group (MR + LPS): birds received a diet lacking methionine (0.35%; marked as 60%Met) and were also subjected to LPS; (4) methionine-supplemented group (MS + LPS): birds received a diet rich in methionine (0.75%; marked as 140%Met) and were challenged with LPS. The experiment lasted for 21 days. On days 17, 19, and 21 of the experiment, chickens in the LPS-stimulated groups were administered 1 mg/kg body weight of LPS through intraperitoneal injection [20]. LPS derived from *Escherichia coli* O55:B5 (L2880, Sigma-Aldrich, St. Louis, MO, USA) was used in this study. In contrast, birds in the CON group were injected with an identical volume of sterilized saline solution. It should be noted that the LPS utilized in this study was dissolved in sterilized saline to prepare a 1 mg/mL solution.

The experiment was carried out at the experimental poultry farm of the College of Animal Science, Shanxi Agricultural University. To minimize potential confounding factors and avoid location bias, cages from different treatment groups were randomly distributed within the house. Moreover, the chicken coop was kept at a steady 34 °C for the first three days, after which the temperature was gradually lowered each week beginning on day 4. This incremental reduction continued until the temperature settled at the surrounding ambient level of 22 °C. For the first three days, the birds were exposed to round-the-clock lighting. From day 4 onward, they followed a 20 h light and 4 h dark cycle, which continued until day 21. The feed composition strictly adhered to China’s NY/T 2004 [21] nutritional standards for broiler chickens. Table 1 presents the dietary composition and nutrient content.

The values for metabolizable energy (ME), crude protein (CP), calcium (Ca), available phosphorus, and total phosphorus in the experimental diets were calculated based on established criteria [22]. The amino acid content of the feeds was measured according to the GB/T 18246-2019 method [23]. The dry matter (DM) and crude ash of the feeds were analyzed in accordance with the procedures outlined in GB/T 6435-2014 [24] and GB/T 6438-2007 [25], respectively. The organic matter (OM) content of the feeds was calculated by subtracting the crude ash content from the DM [26]. The determination of CP was performed using the Kjeldahl method specified in GB/T 6432-2018 [27], with a fully automated Kjeldahl nitrogen analyzer (Kjeltec 8500, FOSS, Hilleroed, Denmark). The Ca content was assessed following the GB/T 6436-2018 method [28], which employed the ethylenediaminetetraacetic acid complexometric titration method. The phosphorus content in the diet was measured using the vanadium-molybdenum yellow colorimetric method according to the GB/T 6437-2018 standard [29] and was analyzed with a spectrophotometer (Spectroquant Prove 300, Merck, Darmstadt, Germany).

### 2.3. Growth Performance

As the experiment progressed, the live weight and total feed intake of the broilers were measured on days 1, 17, and 21. On day 21, the birds were weighed 8 h after LPS injection. Additionally, the average daily feed intake (ADFI), average daily gain (ADG), and feed conversion ratio (F/G) were carried out before LPS stimulation (days 1–16), during LPS stimulation (days 17–21), and over the entire trial period (days 1–21).

### 2.4. Sample Collection

On day 21, blood specimens were drawn 8 h post-LPS administration through the subclavian vein, with one bird randomly selected from each replicate. Before slaughter, blood was collected into 5 mL vacuum tubes that lacked anticoagulant and then left to sit for an hour. Following this, the samples were centrifuged at 3500× *g* at 4 °C for 15 min to separate the serum. The resulting serum was then kept at −80 °C until it was required for further testing. After blood collection, the birds were euthanized by cervical dislocation to minimize suffering, with subsequent harvesting of the duodenum, jejunum, ileum, and liver tissues. Thereafter, the tissues were immediately stored in 4% paraformaldehyde for the observation of intestinal morphology and histopathological examination of the liver tissue. Additionally, part of the liver tissue was flash-frozen in liquid nitrogen and stored at −80 °C for subsequent analysis.

### 2.5. Chemical Analysis

Six serum samples per treatment group (*n* = 6) were analyzed individually without pooling. Using an automated biochemistry analyzer (BS-180, Mindray Biomedical Electronics Co., Ltd., Shenzhen, China), the serum levels of alanine transaminase (ALT), aspartate transaminase (AST), total protein (TP), uric acid (UA), glucose (Glu), and gamma-glutamyl transferase (γ-GT) were assessed. Moreover, the levels of corticosterone (CORT) (ml899951; detection range: 50–1600 pg/mL), LPS (ml059937; detection range: 1.25–80 EU/mL), interleukin-1β (IL-1β) (ml059835L; detection range: 20–640 pg/mL), interleukin-6 (IL-6) (ml059839; detection range: 1–65 pg/mL), tumor necrosis factor-α (TNF-α) (ml002790L; detection range: 2.5–80 pg/mL), and interleukin-10 (IL-10) (ml059830L; detection range: 2.5–84 pg/mL) in the serum were quantified using kits from Shanghai Enzyme-Linked Immunosorbent Assay Biotechnology Co., Ltd. (Shanghai, China). Briefly, standards and samples were added to antibody-coated microplates and incubated with biotin-labeled antibodies followed by HRP-conjugated streptavidin. After washing, substrate solution was added for color development, and the reaction was terminated with stop solution. The absorbance was measured at 450 nm, and concentrations were calculated based on the standard curves.

### 2.6. Liver Antioxidant Activity

Liver tissues were collected from six birds per treatment group (*n* = 6), homogenized individually without pooling, and then centrifuged at 3500× *g* at 4 °C for 15 min. The supernatant was collected for further analysis. Antioxidant capacity in the liver was assessed using kits (Nanjing Jiancheng Bioengineering Research Institute, Nanjing, China). The commercial kits were used to measure total antioxidant capacity (T-AOC) (A015-2-1), malondialdehyde (MDA) (A003-1), catalase (CAT) (A007-1-1), superoxide dismutase (SOD) (A001-3-2), and glutathione peroxidase (GSH-Px) (A005-1) levels.

### 2.7. Liver Tissue Pathology Analysis

Briefly, liver samples from six birds per treatment group were stored in 4% paraformaldehyde, cut into 3 μm-thick sections and stained with hematoxylin and eosin (H&E). Inflammatory changes within the tissue sections were examined using a Leica optical microscope (DM2700 M, Leica Microsystems, Wetzlar, Germany). Five sections from each liver sample were examined and scored under light microscopy. Acute liver injury was assessed to establish an overall tissue pathological score using a composite scoring system for inflammation and necrosis [30]. Acute liver injury was evaluated using a composite histological score based on inflammation and necrosis. Lobular inflammation (0, none; 1, mild; 2, moderate; 3, severe), portal inflammation (0, none; 1, mild; 2, moderate; 3, severe), and necrosis (0, none; 1, <10% of hepatic parenchyma; 2, 10–25% of hepatic parenchyma; 3, >25% of hepatic parenchyma) were scored individually.

### 2.8. Intestinal Morphology

Duodenal, jejunal, and ileal tissue samples from six birds per treatment group were fixed in 4% paraformaldehyde for 24 h, then trimmed, dehydrated, paraffin-embedded, sectioned at 5 µm, and stained with hematoxylin and eosin (H&E). Sections were examined under a light microscope at 100× magnification. Villus height (VH), crypt depth (CD), and the V/C ratio were quantified using Image-Pro Plus 6.0. For each bird, three sections were examined per intestinal segment, and 10 villi of similar length were measured per section [31].

### 2.9. Determination of Gene Expression in Liver

Total RNA was extracted from liver tissues using TRIzol reagent, followed by reverse transcription into cDNA using the PrimeScript RT reagent Kit (Takara, Dalian, China). Then, real-time quantitative polymerase chain reaction (RT-qPCR) was performed with the SYBR Premix Ex Taq™ II kit (Takara, Beijing, China), normalizing to the housekeeping gene *β-actin*. The reaction system had a total volume of 20 µL. Primers were designed and synthesized by Sangon Biotech Co., Ltd. (Shanghai, China), and the specific sequences along with accession numbers are presented in Table 2. The expression levels of the target gene were calculated using the 2^−ΔΔCT^ method.

### 2.10. Correlation Assay

The Spearman correlation analysis method was applied to the data sets to determine the relationships among methionine adenosyltransferase (MAT), glycinamide ribonucleotide transformylase (GNMT), S-adenosylhomocysteine hydrolase (AHCY), 5,10-methylenetetrahydrofolate reductase (MTR), cystathionine β-synthase (CBS), and GSH, conducted through the open-access OmicShare tools (http://www.omicshare.com/tools) (accessed on 6 January 2025).

### 2.11. Statistical Analysis

Data were organized in Microsoft Excel 2021 and analyzed using SPSS 22.0 (IBM Corporation, Chicago, IL, USA). Dataset conformity to normal distribution parameters was objectively determined through Shapiro–Wilk hypothesis testing prior to advanced analytics. Data that conformed to normality and homogeneity of variances were statistically analyzed by one-way ANOVA, along with the Tukey HSD multiple comparisons test. For non-normally distributed data, such as liver histological scores, significant differences were determined using the non-parametric Kruskal–Wallis H test, followed by Dunn–Bonferroni test. Results are presented as means ± standard error of the means (**SEM**), with a significance level of *p* < 0.05 indicating statistically significant differences.

## 3. Results

### 3.1. Growth Performance

As illustrated in Table 3, neither LPS, MR + LPS, nor MS+ LPS had a notable impact (*p* > 0.05) on the overall weight or ADFI of broiler chickens. On the other hand, a marked reduction in ADG (*p* = 0.018) was observed in the LPS and MS + LPS groups compared to CON during the LPS challenge period (days 17–21). Furthermore, the F/G was significantly higher in the MR + LPS group prior to LPS exposure (*p* = 0.007, days 1–16) when compared to the other groups; however, no significant differences (*p* > 0.05) were found among the CON, LPS, and MS + LPS cohorts.

### 3.2. Serum Biochemical Parameters

While serum ALT levels showed no statistically significant variation (*p* > 0.05) between the CON and LPS groups, the MR + LPS group demonstrated notably reduced ALT activities (*p* = 0.005) compared to both the LPS and MS+ LPS groups. Furthermore, AST levels were markedly elevated (*p* = 0.005) in the LPS and MS + LPS groups relative to the CON and MR + LPS groups. While the CON, LPS, and MS + LPS groups exhibited comparable serum TP concentrations (*p* > 0.05), the MR + LPS group showed significantly lower TP levels (*p* = 0.024) when measured against both the CON and LPS groups. Additionally, LDL levels were significantly reduced (*p* = 0.012) in the LPS + MR groups compared with the other groups (Table 4).

### 3.3. Serum Stress and Inflammatory Factors

Table 5 reveals that levels of serum LPS, CORT, IL-1β, and IL-6 were markedly reduced (*p* < 0.001) in the CON, MR + LPS, and MS+ LPS groups compared to the LPS group. While the levels of these markers were significantly lower in the MS + LPS group (*p* < 0.001) than those in the CON group, there was no statistically significant difference (*p* > 0.05) between the MR + LPS and CON groups. Conversely, serum IL-10 concentrations were notably higher (*p* < 0.001) in the CON, MR + LPS, and MS + LPS groups relative to the LPS group. Interestingly, IL-10 levels in the MR + LPS and MS+ LPS groups were significantly higher than those in the CON group significantly (*p* < 0.001). Serum TNF-α levels were significantly decreased (*p* < 0.001) in the CON, MR + LPS, and MS + LPS groups when compared to the LPS group. Although no significant difference (*p* > 0.05) was observed between the MR + LPS and MS + LPS groups for TNF-α, both groups presented a marked reduction (*p* < 0.001) relative to CON.

### 3.4. Liver Antioxidant Capacity

T-AOC levels in the liver were notably higher (*p* = 0.002) in the CON and MR + LPS groups compared to those in the LPS and MS+ LPS groups (Table 6). The hepatic MDA content in the LPS group was significantly higher than that in the other three groups (*p* < 0.001). CAT activity presented a marked increase (*p* = 0.025) in the CON and MR + LPS groups compared to the LPS group, although there was no statistically significant difference (*p* > 0.05) among the CON, LPS, and MS + LPS groups. As for SOD activity, no significant variation (*p* > 0.05) was observed between CON and LPS, but the MR + LPS group exhibited a notably higher activity (*p* = 0.006) than both CON and LPS.

**Figure 1:**
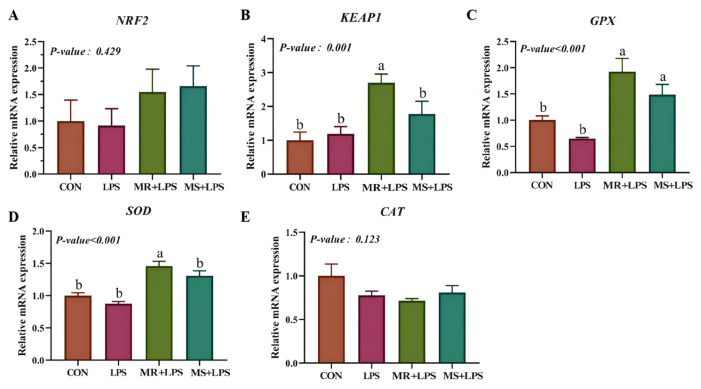
Impact of methionine reduction and supplementation on the expression of antioxidant genes in the liver. (**A**–**E**) Expression of Nuclear factor erythroid 2-related factor 2 (*NRF2*), kelch-like eh-associated protein 1 (*KEAP1*), superoxide dismutase (*SOD*), glutathione peroxidase (*GPX*) and catalase (*CAT*) genes in the liver. CON, birds received a basal diet (0.55%; marked as 100%Met) without LPS challenge; LPS, birds received a basal diet (0.55%; marked as 100%Met) but were exposed to LPS; MR + LPS, birds received a diet lacking methionine (0.35%; marked as 60%Met) and were also subjected to LPS; MS + LPS, birds received a diet rich in methionine (0.75%; marked as 140%Met) and were challenged with LPS. The mRNA expression levels were quantified using the 2^−ΔΔCt^ method and are presented as relative expression levels normalized to the reference gene. Statistical significance was analyzed using one-way ANOVA followed by the Tukey test. *n* = 6/group. ^a,b^ Different superscripts in the same row denote statistically significant differences at *p* < 0.05.

This finding was further corroborated by qPCR analysis (Figure 1). The liver exhibited markedly elevated KEAP1 levels (*p* = 0.021) in the MR + LPS group compared to all other experimental groups. Notably, both the MR + LPS and MS + LPS groups presented substantially greater SOD expression (*p* = 0.001) relative to the CON and LPS groups. Furthermore, *GPX* gene expression demonstrated a significant upregulation (*p* = 0.001) in the MR + LPS group when measured against the CON and LPS groups.

### 3.5. Liver Histopathological Score

**Figure 2:**
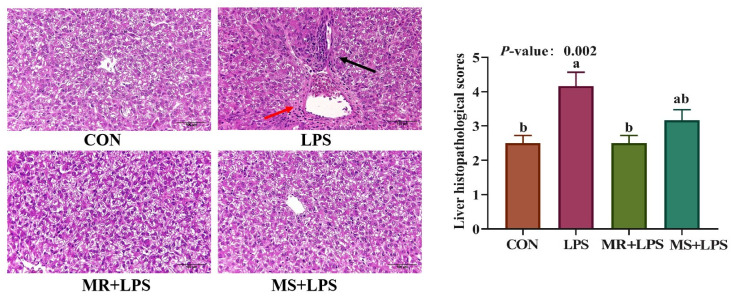
Impact of methionine reduction and supplementation on histopathological analysis of liver in broilers (400×). The black arrows point to inflammatory cells and the red arrows point to the vacuolar degeneration. Statistical significance was analyzed using the non-parametric Kruskal–Wallis H test, followed by Dunn’s T3 post hoc test. *n* = 6/group. CON, birds received a basal diet (0.55%; marked as 100%Met) without LPS challenge; LPS, birds received a basal diet (0.55%; marked as 100%Met) but were exposed to LPS; MR + LPS, birds received a diet lacking methionine (0.35%; marked as 60%Met) and were also subjected to LPS; MS + LPS, birds received a diet rich in methionine (0.75%; marked as 140%Met) and were challenged with LPS. ^a,b^ Different superscripts in the same row denote statistically significant differences at *p* < 0.05.

In Figure 2, the histopathological score of the liver tissue was notably elevated in the LPS group compared to the CON and MR + LPS groups, with a statistically significant difference (*p* = 0.002). Conversely, there was no appreciable distinction (*p* > 0.05) in scores between the LPS and MS + LPS groups. Notably, liver tissues in the LPS group showed extensive necrosis and moderate vacuolization, along with varying levels of inflammatory cell infiltration in the adjacent areas.

### 3.6. Liver Expression of Methionine Metabolizing Enzymes

**Figure 3:**
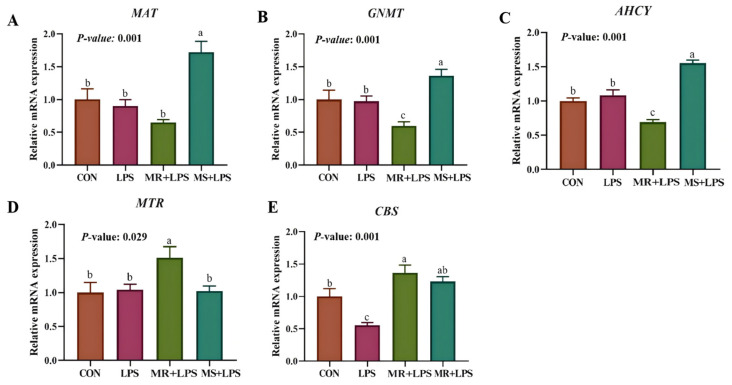
Impact of methionine reduction and supplementation on gene expression of methionine metabolic enzymes in the liver. (**A**–**E**) Expression of methionine adenosyltransferase (*MAT*), glycinamide ribonucleotide transformylase (*GNMT*), s-adenosylhomocysteine hydrolase (*AHCY*), 5,10-methylenetetrahydrofolate reductase (*MTR*), cystathionine β-synthase (*CBS*) genes in the liver. CON, birds received a basal diet (0.55%; marked as 100%Met) without LPS challenge; LPS, birds received a basal diet (0.55%; marked as 100%Met) but were exposed to LPS; MR + LPS, birds received a diet lacking methionine (0.35%; marked as 60%Met) and were also subjected to LPS; MS + LPS, birds received a diet rich in methionine (0.75%; marked as 140%Met) and were challenged with LPS. The mRNA expression levels were quantified using the 2^−ΔΔCt^ method and are presented as relative expression levels normalized to the reference gene. Statistical significance was analyzed using one-way ANOVA followed by the Tukey test. *n* = 6/group. ^a–c^ Different superscripts in the same row denote statistically significant differences at *p* < 0.05.

**Figure 4:**
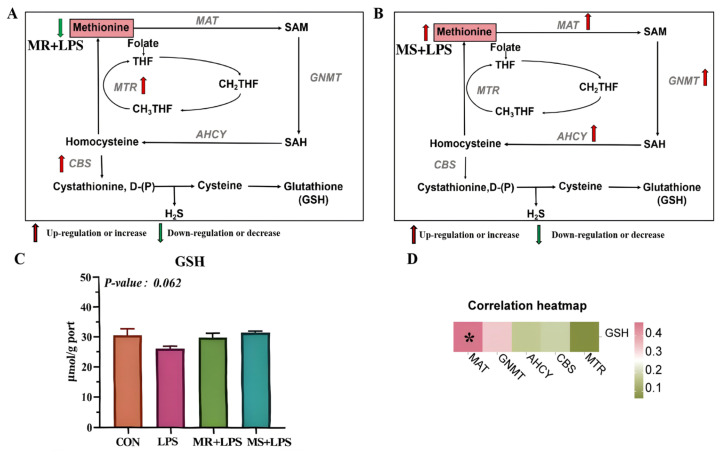
Impact of methionine reduction and supplementation on liver methionine metabolites and enzymes. (**A**) The Pathway illustrating the metabolism of Met to glutathione (GSH) in the MR + LPS group; (**B**) The Pathway illustrating the metabolism of Met to GSH in the MS + LPS group; (**C**) The content of GSH in the liver; (**D**) The figure illustrates methionine adenosyltransferase (*MAT*), glycinamide ribonucleotide transformylase (*GNMT*), S-adenosylhomocysteine hydrolase (*AHCY*), 5,10-methylenetetrahydrofolate reductase (*MTR*), cystathionine β-synthase (*CBS*), and GSH correlations. CON, birds received a basal diet (0.55%; marked as 100%Met) without LPS challenge; LPS, birds received a basal diet (0.55%; marked as 100%Met) but were exposed to LPS; MR + LPS, birds received a diet lacking methionine (0.35%; marked as 60%Met) and were also subjected to LPS; MS + LPS, birds received a diet rich in methionine (0.75%; marked as 140%Met) and were challenged with LPS. Statistical significance was analyzed using one-way ANOVA followed by the Tukey test. *n* = 6/group. * Correlations deemed significant are within the range of 0.01 to 0.05, with red denoting positive correlations and green indicating negative correlations.

*MAT*, *GNMT*, and *AHCY* expression levels in the liver were markedly elevated (*p* = 0.001) in the MS + LPS group compared with the other three groups (Figure 3). Moreover, *CBS* mRNA levels were markedly elevated (*p* = 0.001) in the MR + LPS group compared with the CON and LPS groups. A schematic representation of the methionine metabolic pathway in the MS + LPS and MR + LPS groups, from methionine to GSH, is presented in Figure 4A,B [32].

### 3.7. Methionine Metabolites in the Liver

LPS stimulation, methionine reduction, and methionine supplementation did not significantly affect GSH levels (Figure 4C).

### 3.8. Correlation Assay

Statistical analysis of *MAT*, *GNMT*, *AHCY*, *MTR*, *CBS*, and GSH levels demonstrated a strong positive association between *MAT* and GSH (*p* < 0.05; Table 7). Inverse correlations were observed between glutathione and GNMT, AHCY, MTR, and CBS; however, the relationships were not statistically significant (Figure 4D; *p* > 0.05).

### 3.9. Intestinal Morphology

Table 7 clearly shows that the duodenal V/C ratio was notably elevated in the CON and MS+ LPS cohorts compared to the LPS and MR + LPS groups, with a statistical significance (*p* = 0.038). Moreover, in the jejunum, the CD level was dramatically higher in birds from the MR + LPS group than those from the other three groups (*p* = 0.002). In contrast, CD (*p* = 0.001) and VH (*p* = 0.028) were substantially reduced in the ileums of birds from the MR + LPS and MS + LPS groups when compared with the LPS group.

## 4. Discussion

Increases and decreases in methionine levels can affect the growth performance of poultry [33]. Zhang et al. (2024) found that 0.31% methionine diets notably increased the F/G ratio in Hyline Grey layer chicks compared with 0.54% methionine [34]. Similarly, in this study, broilers in the MR + LPS group (0.35% methionine) exhibited a significantly higher F/G ratio than those in the CON group (0.55% methionine). These findings demonstrate that methionine reduction reduces broiler growth under normal conditions, which may be associated with reduced muscle protein deposition and impaired antioxidant capacity when dietary methionine levels are reduced [35,36]. Previous studies have shown that LPS stimulation serves as an effective immune stress model that induces the production of pro-inflammatory factors in various organs of broilers, compromising intestinal barrier function and decreasing feed intake [37,38]. Our findings demonstrated significantly reduced ADG in the LPS group compared with the CON group under LPS challenge (days 17–21), indicating that the LPS acute stress model was successfully established. From days 17 to 21, neither the MR + LPS nor the MS + LPS groups differed from the LPS group in ADG. Compared with the CON group, the MS+ LPS group showed a significant reduction in ADG, whereas the MR + LPS group did not differ from the CON group. Furthermore, broilers in the MR + LPS group achieved similar ADG compared with those in the CON group (days 17–21). Conversely, the ADG in the MS+ LPS group was notably lower than in the CON group. Overall, these findings imply that limiting methionine intake might help counteract the growth performance decline caused by LPS.

ALT is a sensitive biomarker of liver function, with elevated levels typically indicating increased permeability of the hepatocyte membrane or hepatocellular damage [39]. AST is also an important indicator of hepatic health, and increased levels are often associated with liver stress [40]. The MS + LPS group had significantly increased serum ALT activity, indicating a potential liver burden. Conversely, the MR + LPS group exhibited the lowest AST activity among all treatments. Although this reduction was not statistically different from the CON group, AST levels in the MR + LPS group were markedly lower compared with those in the LPS group, suggesting that an appropriate methionine reduction may mitigate hepatic injury. Therefore, the level of methionine should be slightly adjusted based on the health status and stress conditions. Previous studies indicate that LPS injections induce liver function impairment in chickens [41,42]. Our pathological assessments and scoring confirmed LPS-induced hepatic damage in broiler chickens; however, it was mitigated by reducing dietary methionine. Our findings suggest that methionine reduction maintains hepatic integrity in chickens, possibly by enhancing the antioxidant capacity and reducing serum AST and ALT levels, thereby mitigating liver injury.

Oxidative stress plays a critical role in the development of liver injury. Alterations in antioxidant defense systems may reflect the severity of hepatic damage and provide mechanistic insights into liver dysfunction [43]. Previous studies have shown that LPS induces oxidative stress, which subsequently induces ROS production and suppresses the activities of the antioxidant enzymes [1,44]. MDA, the terminal byproduct of free radical-mediated lipid peroxidation cascades, constitutes a biomarker for quantifying oxidative stress in biological systems [45]. Our study confirmed that LPS induces oxidative stress in broiler chickens and that methionine reduction alleviates this stress. Methionine plays a role in maintaining the normal antioxidant status of animals [46,47]. Methionine reduction enhanced T-AOC in the livers of broilers following LPS stimulation, suggesting that methionine reduction may attenuate LPS-induced oxidative stress. Antioxidant enzymes are crucial for reducing the harmful effects of oxidative stress and guarding against further immune-pathological impairment of the tissues [2]. Notably, SOD and CAT activities were significantly upregulated in the MR + LPS group compared with those in the LPS group, effectively countering LPS-induced oxidative stress. Additionally, the mRNA levels of *SOD* and *GPX* genes were significantly higher in the liver of birds in the MR + LPS group than those in the LPS group. Collectively, these results suggest that methionine reduction may reverse the LPS-induced decrease in antioxidant enzyme activity in birds and improve hepatic antioxidant capacity. CBS is a key enzyme in the methionine transsulfuration pathway, which contributes to cellular redox homeostasis and stress defense [48]. Increased CBS expression may enhance hepatic resistance to inflammatory oxidative damage [49,50]. Our study showed that methionine reduction markedly elevated *CBS* levels in the liver, suggesting an enhanced stress defense capacity under LPS-induced immune stress. CBS is a key enzyme in the methionine transsulfuration pathway, which promotes the conversion of homocysteine to cysteine, thereby providing substrates for antioxidant defense [51,52]. Although hepatic GSH levels did not show significant changes, the upregulation of *CBS* and GPX gene expression in the liver suggests enhanced antioxidant potential. Furthermore, analysis of the Nrf2 signaling pathway revealed elevated expression of hepatic *KEAP1*, *GPX*, and *SOD*, indicating the coordinated activation of multiple antioxidant mechanisms. Therefore, the improvement in antioxidant status observed in this study likely results from the combined regulation of multiple antioxidant enzymes and pathways, rather than changes in GSH alone.

LPS exposure triggers oxidative stress and exacerbates inflammatory responses in animal models [53]. A previous study showed that LPS significantly enhanced pro-inflammatory cytokine secretion, suppressing animal growth [54]. Our study demonstrated a significant elevation in serum levels of LPS, CORT, and pro-inflammatory cytokines (TNF-α, IL-1β, and IL-6) along with a notable reduction in anti-inflammatory IL-10 levels in the LPS group (subjected to LPS stimulation) compared with the CON group. This confirmed the successful establishment of the LPS model. Methionine supplementation has been shown to improve animal immune function, antioxidant status, and growth performance under heat stress and other stressful conditions [55]. In contrast, emerging evidence also suggests that methionine reduction may alleviate immunological stress and improve antioxidant capacity and liver health in LPS-challenged broilers, as reflected by reduced serum LPS, CORT, and pro-inflammatory cytokines, along with increased anti-inflammatory IL-10 levels [15]. Consistent with these observations, serum LPS, CORT, IL-1β, IL-6 and TNF-α levels were significantly lower in the MS + LPS and MR + LPS groups than in the LPS group under LPS stimulation. Serum IL-10 was significantly higher in the MS + LPS and MR + LPS groups than in the LPS and CON groups. Collectively, methionine reduction and supplementation may effectively ameliorate the LPS-induced inflammatory response, achieving an inflammatory status comparable to that observed under non-challenged conditions.

Methionine metabolism is finely regulated by three interconnected pathways, including transmethylation, remethylation, and transsulfuration, which collectively enable an organism to adapt to changes in nutrient availability and inflammatory stress [56,57,58]. In the present study, under LPS-induced immune challenge, the significant upregulation of *MAT*, *GNMT*, and *AHCY* in the MS + LPS group indicates an enhanced transmethylation pathway, thereby promoting the synthesis of SAM and the efficient turnover of methyl donors. Previous studies have shown that methionine and its derived methyl donor SAM play important roles in the regulation of inflammatory responses. For example, methionine treatment increases intracellular SAM levels and enhances DNA methylation in macrophages, while suppressing the expression of LPS-induced pro-inflammatory cytokines [59]. Based on these findings, the present results suggest that the improvement of LPS-induced inflammation observed in the MS + LPS group may be closely associated with enhanced transmethylation activity and its regulatory effects on the transcription of immune-related genes.

In contrast, under LPS-induced immune challenge, the significant upregulation of *MTR* and *CBS* in the MR + LPS group indicates activation of both the remethylation reaction and the transsulfuration pathway. Increased *MTR* expression helps maintain methionine homeostasis by enhancing the remethylation of homocysteine [60]. Furthermore, elevated expression of *CBS*, the rate-limiting enzyme of the transsulfuration pathway, promotes the metabolic flux of homocysteine toward cysteine production and subsequent glutathione synthesis [46]. Previous studies have shown that activation of the transsulfuration pathway enhances glutathione synthesis, thereby strengthening antioxidant defenses under conditions of oxidative and inflammatory stress [52,61]. Consistent with these molecular regulatory mechanisms, these findings suggest that the protective effects observed in the MR + LPS group under LPS challenge may be closely associated with transsulfuration-mediated enhancement of antioxidant capacity and the subsequent attenuation of oxidative stress-related inflammatory damage.

Methionine is an essential component of proteins [62] and exerts beneficial effects on the regulation of intestinal balance, thereby promoting gut health and preventing damage. Beaumont and Blachier (2020) identified adequate methionine as a critical factor for maintaining intestinal health [63]. Gong et al. (2023) highlighted the complexity of methionine-mediated regulation of intestinal morphology, and our results further demonstrate that these effects vary markedly among intestinal segments under LPS stimulation [64]. Additionally, the requirement for methionine varies across different dimensions of intestinal health. For example, the duodenum’s V/C ratio remained high in the MS + LPS group, thus protecting against LPS-induced injury. In contrast, the MR + LPS group in the jejunum demonstrated a significant increase in CD, suggesting that deeper crypts may reflect the body’s adaptive response to LPS, potentially enhancing its defensive capacity through modulation of crypt architecture [65].

A limitation of this study is that methionine restriction and supplementation groups without LPS challenge were not included. Consequently, the independent effects of dietary methionine levels cannot be fully distinguished from those caused by LPS stimulation. Future studies incorporating these treatment groups would help better clarify their respective contributions. Although the present study demonstrated that short-term methionine modulation can alleviate LPS-induced stress, further studies with extended pre-feeding and recovery periods are warranted to assess its long-term adaptability and practical applicability in poultry production. In addition, under LPS challenge, a moderate reduction in dietary methionine appeared to promote the activation of the remethylation and transsulfuration pathways. However, whether this response contributes to the maintenance of methionine metabolic homeostasis requires further investigation, particularly through the measurement of methionine metabolic intermediates.

## 5. Conclusions

This study confirmed the detrimental effects of LPS on broiler chickens and demonstrated that a moderate methionine reduction under LPS challenge promotes the activation of the remethylation and transsulfuration pathways, thereby enhancing cellular antioxidant capacity. This metabolic pathway upregulated the expression and activity of antioxidant enzymes, attenuated LPS-induced oxidative stress and inflammatory responses, and ultimately mitigated liver injury while partially restoring growth performance. In contrast, under inflammatory stress conditions, methionine supplementation mainly exerts anti-inflammatory effects by enhancing transmethylation metabolism and methylation-related regulation. Overall, both strategies adapt to LPS challenge through the activation of different metabolic pathways.

## Figures and Tables

**Table 1:** Composition and nutrient levels of diets (air-dry basis).

Items ^4^	Treatment Groups ^1^			
	CON	LPS	MR + LPS	MS + LPS
Ingredients (%)				
Corn	54.70	54.70	54.70	54.70
Soybean meal	33.50	33.50	33.50	33.50
Corn protein powder	3.00	3.00	3.00	3.00
Wheat flour	2.00	2.00	2.00	2.00
Soybean oil	2.40	2.40	2.40	2.40
Dicalcium phosphate	1.40	1.40	1.40	1.40
Limestone	1.35	1.35	1.35	1.35
Lys	0.35	0.35	0.35	0.35
NaCl	0.30	0.30	0.30	0.30
Choline chloride, 50%	0.20	0.20	0.20	0.20
Thr	0.11	0.11	0.11	0.11
Arginine, 98.5%	0.04	0.04	0.04	0.04
Trace mineral ^2^	0.20	0.20	0.20	0.20
Vitamin permix ^3^	0.03	0.03	0.03	0.03
DL-Met, 99%	0.20	0.20	0.00	0.40
Phytase	0.01	0.01	0.01	0.01
Zeolite powder	0.22	0.22	0.42	0.02
Total	100.00	100.00	100.00	100.00
Calculated nutrient levels (%)				
ME (MJ/kg)	12.47	12.47	12.47	12.47
CP	21.80	21.80	21.80	21.80
Ca	0.90	0.90	0.90	0.90
Available phosphorus	0.35	0.35	0.35	0.35
Total phosphorus	0.67	0.67	0.67	0.67
Measured nutrient levels (%)				
Lys	1.46	1.46	1.46	1.46
Met	0.55	0.55	0.35	0.75
Thr	0.92	0.92	0.92	0.92
Trp	0.26	0.26	0.26	0.26
OM	78.14	78.92	78.59	78.58
CP	21.57	22.21	21.01	21.37
Ca	1.00	1.01	0.98	1.03
Phosphorus	0.53	0.53	0.56	0.60

^1^ CON, birds received a basal diet (0.55%; marked as 100%Met) without LPS challenge; LPS, birds received a basal diet (0.55%; marked as 100%Met) but were exposed to LPS; MR + LPS, birds received a diet lacking methionine (0.35%; marked as 60%Met) and were also subjected to LPS; MS+ LPS, birds received a diet rich in methionine (0.75%; marked as 140%Met) and were challenged with LPS. ^2^ The dietary mineral blend contained: 8 mg Cu, 75 mg Zn, 80 mg Fe, 100 mg Mn, 0.15 mg Se, and 0.35 mg I per kilogram. ^3^ The vitamin premix supplied the following nutrients per kilogram of feed: 15,000 IU of Vitamin A, 3600 IU of Vitamin D_3_, 3 mg of Vitamin K, 2.4 mg of Vitamin B_1_, 9.6 mg of Vitamin B_2_, 3.6 mg of Vitamin B_6_, 0.03 mg of Vitamin B_12_, 30 IU of Vitamin E, 0.15 mg of biotin, 1.5 mg of folic acid, 13.8 mg of pantothenic acid, and 45 mg of niacin. ^4^ ME, metabolizable energy; CP, crude protein; Ca, calcium; Lys, Lysine; Met, Total Methionine; Thr, Threonine; Trp, Tryptophan; OM, organic matter.

**Table 2:** The list of primer pairs for broiler genes used in quantitative real-time PCR.

Gene ^2^	Primers Sequence ^1^ (5′-3′)	Accession No.	Size (bp)
*β-actin*	F: GCTACAGCTTCACCACCACA	NM_205518.2	90
	R: TCTCCTGCTCGAAATCCAGT		
*NRF2*	F: TTCGCAGAGCACAGATACTTC	NM_205117.1	188
	R: TGGGTGGCTGAGTTTGATTAG		
*KEAP1*	F: CTGCTGGAGTTCGCCTACAC	XM_025145847.1	181
	R: CACGCTGTCGATCTGGTACA		
*SOD*	F: GGTCATCCACTTCCAGCAGCAG	U28407.1	379
	R: TAAACGAGGTCCAGCATTTCCAGTTAG		
*CAT*	F: TTGCTATACGGTTCTCCACTGTTGC	NM_001031215.2	255
	R: GTAAAGACTCAGGGCGAAGACTCAAG		
*GPX*	F: GACCAACCCGCAGTACATCA	NM_001277853.3	204
	R: GAGGTGCGGGCTTTCCTTTA		
*MAT*	F: ACATTCAGGAGAATGGAGCGGTCA	NM_001199519.1	87
	R: TCTCCAGCGTAACGGTTTCATCGT		
*GNMT*	F: TCACGGCACTGCTGAAAG	XM_015283546.1	119
	R: CTTGACGACGTGGATGAAGTAG		
*AHCY*	F: CACTCTGGCTCTGGGAATTAG	XM_417331.5	100
	R: CCCACTCTTCATGGGACATTAG		
*MTR*	F: TATGCTGTTGAAGAGGCAGTGGGA	NM_001031104.1	157
	R: AGGCCGAAGTAGTCACAAACGCCA		
*CBS*	F: GGAGAAGCTGATGTCAGAGAAG	XM_416752.4	126
	R: CCACCAGGGAGGAAGTATTTG		

^1^ F, forward primer; R, reverse primer. ^2^ *NRF2*, nuclear factor erythroid 2-related factor 2; *KEAP1*, kelch-like eh-associated protein 1; *SOD*, superoxide dismutase; *CAT*, catalase; *GPX*, glutathione peroxidase; *MAT*, methionine adenosyltransferase; *GNMT*, glycinamide ribonucleotide transformylase; *AHCY*, s-adenosylhomocysteine hydrolase; *MTR*, 5,10-methylenetetrahydrofolate reductase; *CBS*, cystathionine β-synthase.

**Table 3:** Impact of methionine reduction and supplementation on growth performance of broilers.

Items ^3^	Treatment Groups ^1^				SEM	*p*-Value ^2^
	CON	LPS	MR + LPS	MS + LPS		
BW, g						
1 d	38.52	39.16	38.87	39.02	0.144	0.501
17 d	502.31	520.63	429.69	507.82	15.025	0.112
21 d	730.82	704.88	635.14	629.86	14.857	0.115
ADFI, g/d						
1–16 d	36.99	38.16	33.52	36.46	0.874	0.306
17–21 d	87.98	78.16	77.87	85.59	2.797	0.526
1–21 d	49.04	48.07	44.07	48.11	0.974	0.298
ADG, g/d						
1–16 d	28.21	28.85	24.36	28.15	0.793	0.162
17–21 d	57.13 ^a^	46.06 ^b^	51.36 ^ab^	46.26 ^b^	1.636	0.018
1–21 d	32.55	31.43	28.41	30.87	0.672	0.155
F/G, g/g						
1–16 d	1.31 ^b^	1.32 ^b^	1.38 ^a^	1.30 ^b^	0.011	0.007
17–21 d	1.54	1.69	1.51	1.85	0.054	0.091
1–21 d	1.51	1.53	1.55	1.56	0.016	0.710

^1^ CON, birds received a basal diet (0.55%; marked as 100%Met) without LPS challenge; LPS, birds received a basal diet (0.55%; marked as 100%Met) but were exposed to LPS; MR + LPS, birds received a diet lacking methionine (0.35%; marked as 60%Met) and were also subjected to LPS; MS + LPS, birds received a diet rich in methionine (0.75%; marked as 140%Met) and were challenged with LPS. ^2^ Statistical significance was analyzed using one-way ANOVA followed by the Tukey test. *n* = 6/group. ^3^ BW, body weight; ADFI, average daily feed intake; ADG, average daily gain; F/G, feed conversion ratio; SEM, standard error of the means. ^a,b^ Different superscripts in the same row denote statistically significant differences at *p* < 0.05.

**Table 4:** Impact of methionine reduction and supplementation on serum biochemical parameters of broilers.

Items ^3^	Treatment Groups ^1^				SEM	*p*-Value ^2^
	CON	LPS	MR + LPS	MS + LPS		
ALT, ng/L	3.43 ^bc^	4.52 ^ab^	3.03 ^c^	5.08 ^a^	0.252	0.005
AST, ng/L	268.78 ^b^	309.22 ^a^	272.27 ^b^	307.47 ^a^	5.804	0.005
UA, μmol/L	451.55	439.58	427.08	438.65	24.067	0.990
GLO, g/L	17.98	17.86	15.67	16.53	0.462	0.232
TP, g/L	28.42 ^a^	28.97 ^a^	24.73 ^b^	27.10 ^ab^	0.560	0.024
γ-GT, U/L	14.40	12.50	11.97	14.10	0.644	0.489
Glu, mmol/L	12.81	12.97	12.41	13.38	0.278	0.693
TC, mmol/L	2.91	2.91	2.52	2.87	0.065	0.088
TG, mmol/L	0.90	0.86	0.98	0.88	0.030	0.555
HDL, mmol/L	1.92	1.86	1.67	1.99	0.044	0.055
LDL, mmol/L	0.65 ^a^	0.69 ^a^	0.48 ^b^	0.62 ^a^	0.026	0.012

^1^ CON, birds received a basal diet (0.55%; marked as 100%Met) without LPS challenge; LPS, birds received a basal diet (0.55%; marked as 100%Met) but were exposed to LPS; MR + LPS, birds received a diet lacking methionine (0.35%; marked as 60%Met) and were also subjected to LPS; MS + LPS, birds received a diet rich in methionine (0.75%; marked as 140%Met) and were challenged with LPS. ^2^ Statistical significance was analyzed using one-way ANOVA followed by the Tukey test. *n* = 6/group. ^3^ ALT, alanine aminotransferase; AST, aspartate aminotransferase; GLO, globulin; TP, total protein; γ-GT, γ-glutamyl transpeptidase; UA, uric acid; Glu, glucose; TC, total cholesterol; TG, triglyceride; HDL, high density lipoprotein; LDL, low density lipoprotein; SEM, standard error of the means. ^a–c^ Different superscripts in the same row denote statistically significant differences at *p* < 0.05.

**Table 5:** Impact of methionine reduction and supplementation on serum stress and inflammatory factors in broilers.

Items ^3^	Treatment Groups ^1^				SEM	*p*-Value ^2^
	CON	LPS	MR + LPS	MS + LPS		
LPS, EU/L	269.02 ^b^	305.89 ^a^	253.24 ^b^	230.01 ^c^	5.959	<0.001
CORT, ng/L	423.22 ^b^	484.38 ^a^	413.72 ^b^	379.77 ^c^	7.768	<0.001
IL-1β, ng/L	136.72 ^b^	155.28 ^a^	132.31 ^bc^	123.75 ^c^	2.615	<0.001
IL-6, ng/L	52.63 ^b^	63.60 ^a^	49.87 ^b^	43.20 ^c^	1.496	<0.001
IL-10, ng/L	57.30 ^c^	45.50 ^d^	61.90 ^b^	67.81 ^a^	1.679	<0.001
TNF-α, ng/L	71.58 ^b^	81.98 ^a^	67.57 ^c^	63.84 ^c^	1.423	<0.001

^1^ CON, birds received a basal diet (0.55%; marked as 100%Met) without LPS challenge; LPS, birds received a basal diet (0.55%; marked as 100%Met) but were exposed to LPS; MR + LPS, birds received a diet lacking methionine (0.35%; marked as 60%Met) and were also subjected to LPS; MS + LPS, birds received a diet rich in methionine (0.75%; marked as 140%Met) and were challenged with LPS. ^2^ Statistical significance was analyzed using one-way ANOVA followed by the Tukey test. *n* = 6/group. ^3^ LPS, lipopolysaccharide; CORT, corticosterone; IL-6, interleukin-6; IL-1β, interleukin-1β; IL-10, interleukin-10; TNF-α, tumor necrosis factor-α; SEM, standard error of the means. ^a–d^ Different superscripts in the same row denote statistically significant differences at *p* < 0.05.

**Table 6:** Impact of methionine reduction and supplementation on liver antioxidant activity in broilers.

Items ^3^	Treatment Groups ^1^				SEM	*p*-Value ^2^
	CON	LPS	MR + LPS	MS + LPS		
T-AOC, U/mg prot	1.22 ^a^	0.67 ^b^	1.16 ^a^	0.80 ^b^	0.067	0.002
MDA, nmol/mg prot	0.32 ^b^	0.72 ^a^	0.40 ^b^	0.36 ^b^	0.042	<0.001
CAT, U/mg prot	8.43 ^a^	6.28 ^b^	8.52 ^a^	7.17 ^b^	0.315	0.002
SOD, U/mg prot	5.70 ^b^	5.85 ^b^	8.16 ^a^	5.97 ^b^	0.310	0.006
GSH-Px, U/mg prot	32.71	45.22	43.84	36.83	4.272	0.725

^1^ CON, birds received a basal diet (0.55%; marked as 100%Met) without LPS challenge; LPS, birds received a basal diet (0.55%; marked as 100%Met) but were exposed to LPS; MR + LPS, birds received a diet lacking methionine (0.35%; marked as 60%Met) and were also subjected to LPS; MS + LPS, birds received a diet rich in methionine (0.75%; marked as 140%Met) and were challenged with LPS. ^2^ Statistical significance was analyzed using one-way ANOVA followed by the Tukey test. *n* = 6/group. ^3^ T-AOC, total antioxidant capacity; MDA, malondialdehyde; CAT, catalase; SOD, superoxide dismutase; GSH-Px, glutathione peroxidase; prot, total protein; SEM, standard error of the means. ^a,b^ Different superscripts in the same row denote statistically significant differences at *p* < 0.05.

**Table 7:** Impact of methionine reduction and supplementation on intestinal morphology of broilers.

Items ^3^	Treatment Groups ^1^				SEM	*p*-Value ^2^
	CON	LPS	MR + LPS	MS + LPS		
Duodenum						
VH, μm	1345.71	1247.76	1184.45	1288.71	24.673	0.121
CD, μm	130.41	131.90	147.30	121.39	4.177	0.172
V/C	10.39 ^a^	9.74 ^b^	8.22 ^b^	10.67 ^a^	0.341	0.038
Jejunum						
VH, μm	668.80	647.80	650.57	668.63	16.639	0.958
CD, μm	96.24 ^b^	97.14 ^b^	126.61 ^a^	82.00 ^b^	4.762	0.002
V/C	6.96 ^a^	6.79 ^ab^	5.33 ^b^	8.33 ^a^	0.322	0.004
Ileum						
VH, μm	365.12 ^ab^	400.88 ^a^	308.69 ^b^	329.23 ^b^	12.243	0.028
CD, μm	55.35 ^ab^	61.50 ^a^	50.09 ^bc^	46.62 ^c^	1.582	0.001
V/C	6.65	6.61	6.18	7.11	0.229	0.587

^1^ CON, birds received a basal diet (0.55%; marked as 100%Met) without LPS challenge; LPS, birds received a basal diet (0.55%; marked as 100%Met) but were exposed to LPS; MR + LPS, birds received a diet lacking methionine (0.35%; marked as 60%Met) and were also subjected to LPS; MS + LPS, birds received a diet rich in methionine (0.75%; marked as 140%Met) and were challenged with LPS. ^2^ Statistical significance was analyzed using one-way ANOVA followed by the Tukey test. *n* = 6/group. ^3^ VH, villus height; CD, crypt depth; V/C, the ratio villus height to crypt depth; SEM, standard error of the means. ^a–c^ Different superscripts in the same row denote statistically significant differences at *p* < 0.05.

## Data Availability

The datasets used and analyzed during the current study are available upon request from the corresponding author.

## Abbreviations

The following abbreviations are used in this manuscript:

ADFI	Average Daily Feed Intake
ADG	Average daily gain
AHCY	S-Adenosylhomocysteine Hydrolase
ALT	Alanine Aminotransferase
AST	Aspartate Aminotransferase
BW	Body Weight
Ca	Calcium
CAT	Catalase
CBS	Cystathionine β-Synthase
CD	Crypt Depth
CORT	Corticosterone
CP	Crude Protein
F/G	Feed Conversion Ratio
GLO	Globulin
GLU	Glucose
GNMT	Glycinamide Ribonucleotide Transformylase
GPX	Glutathione Peroxidase
GSH	Glutathione
HDL	High Density Lipoprotein
IL-1β	Interleukin-1β
IL-6	Interleukin-6
IL-10	Interleukin-10
KEAP1	Kelch-Like ECH-Associated Protein 1
LDL	Low Density Lipoprotein
LPS	Lipopolysaccharide
Lys	Lysine
MAT	Methionine Adenosyltransferase 1A
ME	Metabolizable Energy
Met	Methionine
MTR	5,10-Methylenetetrahydrofolate Reductase
OM	Organic Matter
ROS	Reactive Oxygen Species
SAH	S-Adenosylhomocysteine
SAM	S-Adenosylmethionine
SOD	Superoxide Dismutase
TC	Total Cholesterol
TG	Triglyceride
Thr	Threonine
TNF-α	Tumor Necrosis Factor-α
TP	Total Protein
Trp	Tryptophan
UA	Uric Acid
V/C	The Ratio Villus Height To Crypt Depth
VH	Villus Height
γ-GT	γ-Glutamyl Transpeptidase

## References

[1] Zheng X.C., Wu Q.J., Song Z.H., Zhang H., Zhang J.F., Zhang L.L., Zhang T.Y., Wang C., Wang T. (2016). Effects of oridonin on growth performance and oxidative stress in broilers challenged with lipopolysaccharide. Poult. Sci..

[2] Zheng Y., Zhang J., Zhou H., Guo Y., Ma Q., Cheng J. (2020). Effects of dietary pyrroloquinoline quinone disodium supplementation on inflammatory responses, oxidative stress, and intestinal morphology in broiler chickens challenged with lipopolysaccharide. Poult. Sci..

[3] Zhang J., Han H., Zhang L., Wang T. (2021). Dietary bisdemethoxycurcumin supplementation attenuates lipopolysaccharide-induced damages on intestinal redox potential and redox status of broilers. Poult. Sci..

[4] Ishihara T., Tanaka K.-I., Takafuji A., Miura K., Mizushima T. (2023). Attenuation of LPS-induced lung injury by benziodarone via reactive oxygen species reduction. Int. J. Mol. Sci..

[5] Peng S., Hang N., Liu W., Guo W., Jiang C., Yang X., Xu Q., Sun Y. (2016). Andrographolide sulfonate ameliorates lipopolysaccharide-induced acute lung injury in mice by down-regulating MAPK and NF-κB pathways. Acta Pharm. Sin. B.

[6] Xia Y., Wang Y., Xue J., Chu J., Zhang Y., She F., Chen H., Li S. (2025). Naringenin’s rescue of broiler spleen from LPS-triggered pyroptosis and inflammation: Decoding the AMPK/PINK1/Parkin-driven mitophagy pathway. Poult. Sci..

[7] Liu G., Magnuson A.D., Sun T., Tolba S.A., Starkey C., Whelan R., Lei X.G. (2019). Supplemental methionine exerted chemical form-dependent effects on antioxidant status, inflammation-related gene expression, and fatty acid profiles of broiler chicks raised at high ambient temperature. J. Anim. Sci..

[8] Gong L., Xu H., Zhu Q., Mahmood T., Mercier Y., Fu J., Han Q., Guo Y. (2025). Methionine sources and total sulfur amino acids to lysine ratio regulate the inflammatory responses of lipopolysaccharide-challenged broilers. Poult. Sci..

[9] Maynard C.W., Gilbert E., Yan F., Cline M.A., Dridi S. (2023). Peripheral and central impact of methionine source and level on growth performance, circulating methionine levels and metabolism in broiler chickens. Animals.

[10] Magnuson A.D., Liu G., Sun T., Tolba S.A., Xi L., Whelan R., Lei X.G. (2020). Supplemental methionine and stocking density affect antioxidant status, fatty acid profiles, and growth performance of broiler chickens. J. Anim. Sci..

[11] Yang Y., Xu Y., Shi Y., Li B., Xie Y., Le G. (2024). Dietary methionine supplementation improves cognitive dysfunction associated with transsulfuration pathway upregulation in mouse models of subacute aging. Res. Sq..

[12] Montout L., Poullet N., Bambou J.C. (2021). Systematic review of the interaction between nutrition and immunity in livestock: Effect of dietary supplementation with synthetic amino acids. Animals.

[13] Pan Y., Fu M., Chen X., Guo J., Chen B., Tao X. (2020). Dietary methionine restriction attenuates renal ischaemia/reperfusion-induced myocardial injury by activating the CSE/H_2_S/ERS pathway in diabetic mice. J. Cell. Mol. Med..

[14] Wu G., Han L., Shi Y., Feng C., Yan B., Sun J., Tang X., Le G. (2020). Effect of different levels of dietary methionine restriction on relieving oxidative stress and behavioral deficits in middle-aged mice fed low-, medium-, or high-fat diet. J. Funct. Foods.

[15] Pang X., Miao Z., Dong Y., Cheng H., Xin X., Wu Y., Han M., Su Y., Yuan J., Shao Y. (2023). Dietary methionine restriction alleviates oxidative stress and inflammatory responses in lipopolysaccharide-challenged broilers at early age. Front. Pharmacol..

[16] Lugata J.K., Ndunguru S.F., Reda G.K., Gulyás G., Knop R., Oláh J., Czeglédi L., Szabó C. (2024). In ovo feeding of methionine affects antioxidant status and growth-related gene expression of TETRA SL and Hungarian indigenous chicks. Sci. Rep..

[17] Teng P.Y., Liu G., Choi J., Yadav S., Wei F., Kim W.K. (2023). Effects of levels of methionine supplementations in forms of L- or DL-methionine on the performance, intestinal development, immune response, and antioxidant system in broilers challenged with *Eimeria* spp.. Poult. Sci..

[18] Martín D., Ordás M.C., Carvalho I., Díaz-Rosales P., Nuñez-Ortiz N., Vicente-Gil S., Arrogante A., Zarza C., Machado M., Costas B. (2023). L-methionine supplementation modulates IgM^+^ B cell responses in rainbow trout. Front. Immunol..

[19] Chen J., Yang W., Liu H., Niu J., Liu Y., Cheng Q. (2024). Protective effect of Macleaya cordata isoquinoline alkaloids on lipopolysaccharide-induced liver injury in broilers. Anim. Biosci..

[20] Wu Y., Zhang Y., Zhou M., Liu P., Rao X., Zhang Y., Mi M. (2025). Methionine ameliorates intestinal injury in senescence-accelerated mouse prone-8 mice by reducing sulfate-reducing bacteria and enhancing barrier function. Front. Nutr..

[21] (2004). Feeding Standard of Chicken.

[22] Xiong B., Luo Q., Zheng S., Zhao F., Zheng S. (2020). Chinese Feed Composition and Nutritive Value Table (31st ed., 2020). China Feed.

[23] (2019). Determination of Amino Acids in Feed.

[24] (2014). Determination of Moisture in Feed.

[25] (2007). Determination of Crude Ash in Feed.

[26] Hu J., Zhang S., Li M., Zhao G. (2024). Impact of dietary supplementation with β-alanine on the rumen microbial crude protein supply, nutrient digestibility and nitrogen retention in beef steers elucidated through sequencing the rumen bacterial community. Anim. Nutr..

[27] (2014). Determination of Crude Protein in Feed—Kjeldahl Method.

[28] (2018). Determination of Calcium in Feed.

[29] (2018). Determination of Phosphorus in Feed.

[30] Siegmund B., Lear-Kaul K.C., Faggioni R., Fantuzzi G. (2002). Leptin deficiency, not obesity, protects mice from Con A-induced hepatitis. Eur. J. Immunol..

[31] Zhao Y., Liu C., Niu J., Cui Z., Zhao X., Li W., Zhang Y., Yang Y., Gao P., Guo X. (2023). Impacts of dietary fiber level on growth performance, apparent digestibility, intestinal development, and colonic microbiota and metabolome of pigs. J. Anim. Sci..

[32] Sanderson S.M., Gao X., Dai Z., Locasale J.W. (2019). Methionine metabolism in health and cancer: A nexus of diet and precision medicine. Nat. Rev. Cancer.

[33] Savino R.J., Kempisty B., Mozdziak P. (2022). The potential of a protein model synthesized absent of methionine. Molecules.

[34] Zhang J., Geng S., Zhu Y., Li L., Zhao L., Ma Q., Huang S. (2024). Effects of dietary methionine supplementation on the growth performance, immune responses, antioxidant capacity, and subsequent development of layer chicks. Poult. Sci..

[35] Liu G., Choppa V.S.R., Sharma M.K., Ko H., Choi J., Kim W.K. (2024). Effects of methionine supplementation in a reduced protein diet on growth performance, oxidative status, intestinal health, oocyst shedding, and methionine and folate metabolism in broilers under Eimeria challenge. J. Anim. Sci. Biotechnol..

[36] Jespersen J.C., Sommer K.M., White C.S., Froebel L.E., Dorigam J.C.P., Harsh B.N., Dilger R.N. (2024). Effects of a coccidiosis challenge on dietary methionine recommendations in broilers. Poult. Sci..

[37] Bavananthasivam J., Alkie T.N., Matsuyama-Kato A., Hodgins D.C., Sharif S. (2019). Characterization of innate responses induced by in ovo administration of encapsulated and free forms of ligands of toll-like receptor 4 and 21 in chicken embryos. Res. Vet. Sci..

[38] Jiang J., Qi L., Lv Z., Jin S., Wei X., Shi F. (2019). Dietary stevioside supplementation alleviates lipopolysaccharide-induced intestinal mucosal damage through anti-inflammatory and antioxidant effects in broiler chickens. Antioxidants.

[39] Wang J., Song Y., Dou X., Sun J., Yang X., Zhang Y., Liu Z., Li Y., Li H. (2025). Liquid crystal microcavity biosensors for real-time liver injury monitoring via whispering gallery mode laser. Research.

[40] Senior J.R. (2012). Alanine aminotransferase: A clinical and regulatory tool for detecting liver injury—Past, present, and future. Clin. Pharmacol. Ther..

[41] Chen L., Ma S., Cao A., Zhao R. (2024). Bile acids promote lipopolysaccharide clearance via the hepato-biliary pathway in broiler chickens. Ecotoxicol. Environ. Saf..

[42] Jangra A., Rajput P., Dwivedi D.K., Lahkar M. (2020). Amelioration of repeated restraint stress-induced behavioral deficits and hippocampal anomalies with taurine treatment in mice. Neurochem. Res..

[43] Sadasivam N., Kim Y.J., Radhakrishnan K., Kim D.K. (2022). Oxidative stress, genomic integrity, and liver diseases. Molecules.

[44] Surai P.F., Kochish I.I., Fisinin V.I., Kidd M.T. (2019). Antioxidant defence systems and oxidative stress in poultry biology. Antioxidants.

[45] Mohideen K., Chandrasekar K., Ramsridhar S., Rajkumar C., Ghosh S., Dhungel S. (2023). Assessment of oxidative stress by the estimation of lipid peroxidation marker malondialdehyde (MDA) in patients with chronic periodontitis: A systematic review and meta-analysis. Int. Dent. J..

[46] Chen N.N., Liu B., Xiong P.W., Guo Y., He J.N., Hou C.C., Ma L.X., Yu D.Y. (2018). Safety evaluation of zinc methionine in laying hens: Effects on laying performance, clinical blood parameters, organ development, and histopathology. Poult. Sci..

[47] Liu R., Diao Q., Cui K. (2020). Effect of dietary methionine deficiency followed by a re-feeding phase on the hepatic antioxidant activities of lambs. Animals.

[48] Liu N., Lin X., Huang C. (2020). Activation of the reverse transsulfuration pathway through NRF2/CBS confers erastin-induced ferroptosis resistance. Br. J. Cancer.

[49] Xi C., Pang J., Xue W., Cui Y., Jiang N., Zhi W., Shi H., Horuzsko A., Pace B.S., Zhu X. (2025). Transsulfuration pathway activation attenuates oxidative stress and ferroptosis in sickle primary erythroblasts and transgenic mice. Commun. Biol..

[50] Elwan H., Xie C., Miao L.P., Dong X., Zou X.T., Mohany M. (2021). Methionine alleviates aflatoxin B1-induced broiler chicks embryotoxicity through inhibition of caspase-dependent apoptosis and enhancement of cellular antioxidant status. Poult. Sci..

[51] Conter C., Fruncillo S., Fernández-Rodríguez C., Martínez-Cruz L.A., Dominici P., Astegno A. (2020). Cystathionine β-synthase is involved in cysteine biosynthesis and H2S generation in Toxoplasma gondii. Sci. Rep..

[52] Niu W.N., Yadav P.K., Adamec J., Banerjee R. (2015). S-glutathionylation enhances human cystathionine β-synthase activity under oxidative stress conditions. Antioxid. Redox Signal..

[53] Rosadini C.V., Kagan J.C. (2017). Early innate immune responses to bacterial LPS. Curr. Opin. Immunol..

[54] Tan J., Li S., Guo Y., Applegate T.J., Eicher S.D. (2014). Dietary L-arginine supplementation attenuates lipopolysaccharide-induced inflammatory response in broiler chickens. Br. J. Nutr..

[55] Kalvandi O., Sadeghi A., Karimi A. (2019). Methionine supplementation improves reproductive performance, antioxidant status, immunity and maternal antibody transmission in breeder Japanese quail under heat stress conditions. Arch. Anim. Breed..

[56] Bottiglieri T. (2002). S-Adenosyl-L-methionine (SAMe): From the bench to the bedside—Molecular basis of a pleiotropic molecule. Am. J. Clin. Nutr..

[57] Finkelstein J.D., Martin J.J., Harris B.J. (1988). Methionine metabolism in mammals: The methionine-sparing effect of cystine. J. Biol. Chem..

[58] Lu S.C. (2009). Regulation of hepatic glutathione synthesis. Mol. Asp. Med..

[59] Ji J., Xu Y., Zheng M., Luo C., Lei H., Qu H., Shu D. (2019). Methionine attenuates lipopolysaccharide-induced inflammatory responses via DNA methylation in macrophages. ACS Omega.

[60] Blom H.J., Smulders Y. (2011). Overview of homocysteine and folate metabolism, with special reference to cardiovascular disease and neural tube defects. J. Inherit. Metab. Dis..

[61] Mosharov E., Cranford M.R., Banerjee R. (2000). The quantitatively important relationship between homocysteine metabolism and glutathione synthesis by the transsulfuration pathway and its regulation by redox changes. Biochemistry.

[62] Saito Y., Iwatsuki K., Hanyu H., Maruyama N., Aihara E., Tadaishi M., Shimizu M., Kobayashi-Hattori K. (2017). Effect of essential amino acids on enteroids: Methionine deprivation suppresses proliferation and affects differentiation in enteroid stem cells. Biochem. Biophys. Res. Commun..

[63] Beaumont M., Blachier F. (2020). Amino Acids in Intestinal Physiology and Health.

[64] Gong L., Mahmood T., Mercier Y. (2023). Dietary methionine sources and levels modulate the intestinal health status of broiler chickens. Anim. Nutr..

[65] Awad W.A., Ghareeb K., Abdel-Raheem S., Bohm J. (2009). Effects of dietary inclusion of probiotic and synbiotic on growth performance, organ weights, and intestinal histomorphology of broiler chickens. Poult. Sci..

